# GIS–AHP–based land suitability assessment for sustainable agricultural planning on Qeshm Island

**DOI:** 10.1038/s41598-026-63402-5

**Published:** 2026-07-23

**Authors:** Asef Darvishi, Maryam Yousefi, Seyed Mohsen Mousavi, Hadi Khoshnamvand, Faezeh Borhani, Michael Schirrmann

**Affiliations:** 1 Leibniz Institute for Agricultural Engineering and Bioeconomy (ATB), Potsdam-Bornim, Max-Eyth-Allee 100, 14469 Potsdam, Germany; 2 Leibniz Centre for Agricultural Landscape Research (ZALF), Müncheberg, Germany; 3 Department of Environmental Science, Faculty of Natural Resources, University of Gilan, Rasht, Iran; 4 Department of Biodiversity and Ecosystem Management, Environmental Sciences Research Institute, Shahid Beheshti University, G.C., Evin, Tehran, Iran; 5 Department of Environmental Engineering, Faculty of Civil and Environmental Engineering, Tarbiat Modares University, Tehran, 1411713116 Iran

**Keywords:** Landscape ecology, Socio-ecological systems, Biodiversity, Multi-criteria land evaluation, Ecology, Ecology, Environmental sciences, Environmental social sciences

## Abstract

Sustainable agricultural planning necessitates a holistic approach that considers ecological, economic, and social dimensions, balancing agricultural yield with long-term ecological sustainability. In the face of intensifying environmental pressures, such as climate change, land degradation, and biodiversity loss, agriculture must adopt adaptive land use strategies to ensure long-term viability. This paper explores how integrating landscape ecology principles into agricultural planning on Qeshm Island can enhance landscape resilience and efficiency to guide land use strategies that preserve biodiversity and sustain ecosystem services. A land suitability model was applied by converting socio-ecological factors (soil, slope, geology, water accessibility, land use and land cover, and sensitive habitats) into standardized raster layers, weighting them with AHP, and integrating them through a GIS-based weighted overlay. Suitability map was evaluated through accuracy assessment and sensitivity analysis to ensure reliability. The results showed high and very high suitability areas (18,628 ha) aligned with optimal conditions, while moderate zones (13,935 ha) required interventions such as erosion control and water management. Low-suitability (1,408 ha) and unsuitable areas (112,188 ha) faced significant constraints as steep topography and erosion-prone soils, limiting agricultural potential. The findings underscore the importance of interdisciplinary collaboration (e.g., agronomy, social, ecology, and geography) and the adoption of innovative planning tools to achieve sustainable agricultural landscapes.

## Introduction

Agricultural landscapes across Asia are increasingly facing intensifying pressures that jeopardize their ecological sustainability and long-term crop yield productivity^1,2^. In many Asian countries, a significant proportion of agricultural areas are facing increasing trend of partial abandonment, driven by a complex interplay of factors such as soil degradation (biophysical), fragmented landholdings (structural), declining farm income (economic), and insufficient land management policies (policy-related)^3–5^. These pressures often lead to dual processes of intensification and marginalization, resulting in dramatic changes in Land Use and Land Cover (LULC) patterns and contributing to environmental degradation^6,7^.

Similar dynamics (e.g., fragmentation and intensification) can be observed throughout Asia’s rural regions, including those in the Middle East. On Qeshm Island, situated in the Persian Gulf, traditional agricultural practices are shifting to commercial cropping as a result of both external pressures, such as climate change, and internal factors, including water scarcity^8–12^. These changes mirror the broader trends in rural abandonment and LULC intensification across the Middle East, leading to ecological imbalances and a growing gap between ecological capacity and land use demand^13^. Yet, like many rural landscapes, Qeshm Island holds considerable potential for sustainable development, owing to its unique ecological, cultural, and historical values.

One of the most effective science-based approaches for promoting sustainability in rural landscapes is landscape planning^14–17^, grounded in spatial analysis, ecological modeling, and multi-criteria evaluation to integrate ecological, social, and economic considerations. Land use serves as a manifestation of socio-economic and cultural processes over time and space, integrating historical, economic, social, and cultural potentials with the interactions between ecosystem functions and human knowledge^18–20^. By integrating landscape ecology principles into planning processes, we can ecologically optimize spatial organization, LULC, and land protection^21–23^. This approach offers a critical tool for environmental management, helping to align human activities with ecological processes and conservation goals^24–26^.

Landscape ecology principles provide a robust theoretical framework for evaluating, planning, and managing LULC by addressing spatial heterogeneity, ecological sensitivity, and human–environment interactions across landscapes^27^. While fragmentation and connectivity are commonly used indicators in landscape-scale biodiversity assessments, landscape ecology extends beyond the quantitative analysis of spatial metrics and also encompasses the integration of ecological processes, LULC dynamics, and spatial planning^22,28,29^. In this study, landscape ecology principles are operationalized through the integration of ecological constraints, LULC patterns, water accessibility, and environmentally sensitive habitats into a socio-ecological land suitability framework. These principles not only help resolve land use conflicts but also improve aesthetic and recreational value of rural landscapes, which can stimulate recreational demand and highlight the area’s cultural and historical heritage^30,31^. In this context, landscape planning becomes a powerful mechanism for fostering sustainable agricultural systems that balance human needs with environmental stewardship^32–34^. Our study employs a GIS-based multi-criteria evaluation approach to translate these landscape ecology principles into an operational land use planning framework, supported by the Analytic Hierarchy Process (AHP).

AHP is widely used in environmental and land-suitability studies^35–37^. It provides a transparent structure for weighting heterogeneous socio-ecological criteria such as soil characteristics, slope, geology, water accessibility, existing LULC, habitat sensitivity, fragmentation, and connectivity that cannot be compared directly^5^. Recent studies further demonstrate the wide applicability and robustness of GIS–AHP frameworks in land suitability and environmental planning. GIS-based AHP models have been successfully applied to crop-specific suitability assessments, including peach cultivation in northwestern Turkey^38^, canola production as a climate change adaptation strategy^39^, and dragon fruit cultivation in tropical environments^40^. In arid and semi-arid regions, the integration of AHP with GIS has proven effective in prioritizing critical constraints such as water availability, soil properties, and topographic conditions to support efficient agricultural planning^36^. Beyond conventional agricultural contexts, AHP-based spatial evaluation has also been extended to urban agriculture planning^41^ and combined with machine learning approaches to enhance land suitability modeling for sisal production^42^. Collectively, these studies confirm AHP as a transparent, flexible, and reproducible decision-support method for weighting heterogeneous environmental and socio-ecological criteria in complex land use planning scenarios.

This methodological integration allows us to convert landscape ecological concepts into measurable decision layers^43^. It enables a systematic evaluation of agricultural landscape that reflects both ecological processes and human constraints. By combining landscape ecology^44,45^ theory with multi-criteria evaluation approach^46^, we create a replicable and data-driven framework for guiding agricultural planning in sensitive landscapes.

This paper examines application of landscape ecology principles into agricultural planning on Qeshm Island, aiming to optimize land use in ways that preserve biodiversity, maintain ecosystem services, and foster resilient agricultural systems. By examining spatial relationships among ecological sensitivity, LULC patterns, slope gradients, and proximity to water sources within a landscape planning framework, and applying a land suitability model, we propose strategies that align agricultural activities with the island’s distinctive ecological characteristics. Our findings highlight the importance of interdisciplinary (e.g., agronomy, sociology, ecology, and geography) collaboration and innovative planning tools in creating sustainable agricultural landscapes that support both human livelihoods and the environment.

The novelty of this study lies in translating landscape ecology concepts, often discussed at a theoretical level, into a practical, data-driven framework for agricultural land use planning. We calibrated Makhdoom’s ecological capability model^47^ into socio-ecological capability model. The novelty of this study lies in translating landscape ecology concepts into a practical GIS-based planning framework for agricultural land-suitability assessment. Rather than focusing solely on agricultural productivity or isolated biophysical conditions^48^, our framework integrates LULC dynamics, ecological sensitivity, and human-related spatial factors to support environmentally informed agricultural planning in a resource-constrained island.

## Material and methods

### Study area

**Fig. 1:**
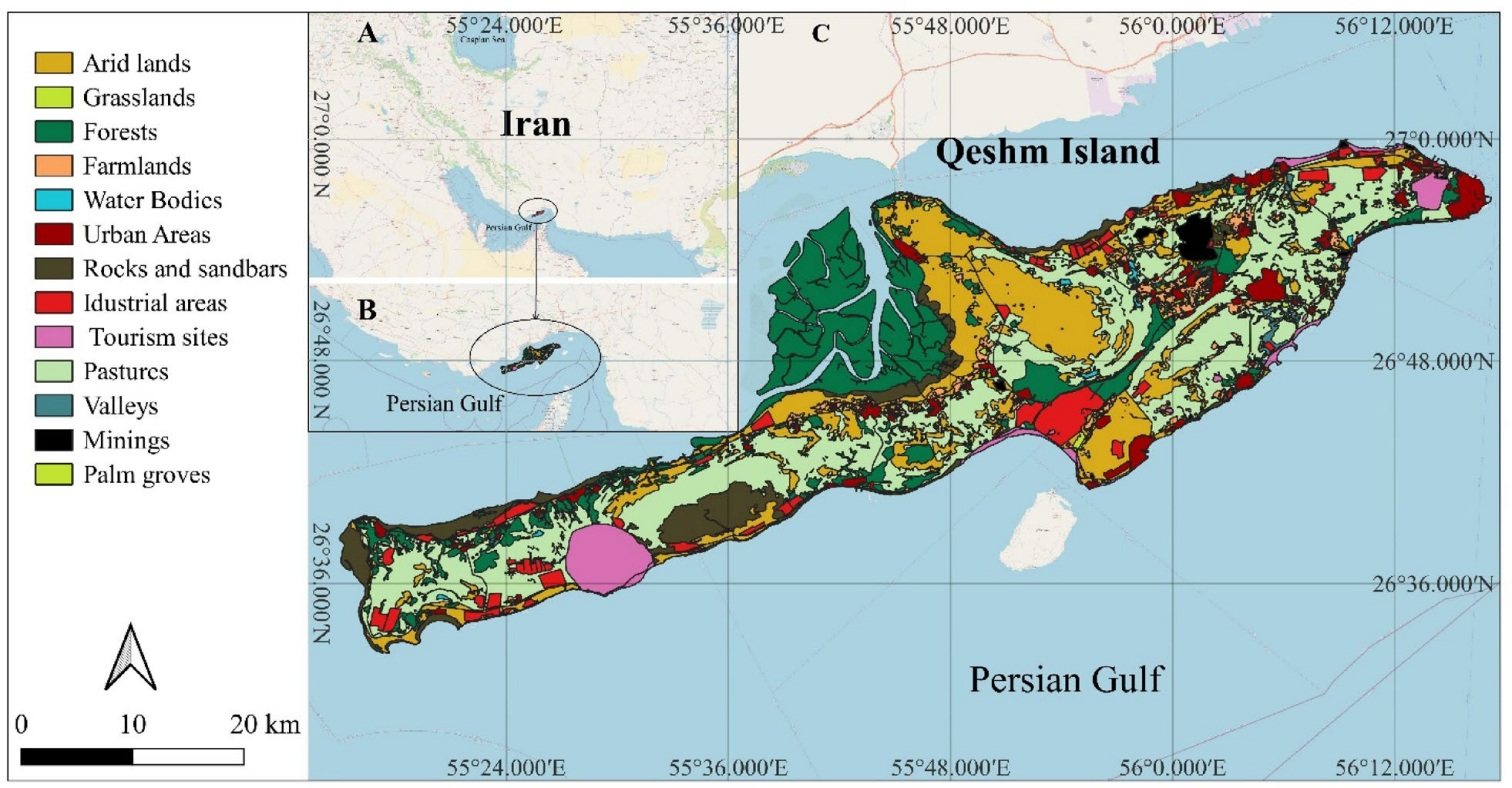
Geographic context of the study area. (**A**) Location of Qeshm Island within Iran; (**B**) Location of Qeshm Island in the Persian Gulf; (**C**) land use and land cover classes of Qeshm Island. The map was created by the authors using ArcGIS Desktop (ArcMap) version 10.8.2 (Esri, Redlands, CA, USA; https://www.esri.com/en-us/arcgis/products/arcgis-desktop/overview.

Qeshm Island, located in the Persian Gulf (Fig. 1), presents a unique case for exploring sustainable agricultural practices within a sensitive ecological framework^49^. This island, with its distinctive biodiversity and fragile ecosystems, is facing increasing pressures from climate change, land use intensification, and shifting socio-economic dynamics^49–55^. The study area, Qeshm Island, is underlain by sedimentary formations including limestones, marls, sandstones, and silty deposits, overlain by Quaternary alluvial, dune, and terrace sediments. Soils are primarily shallow and arid, including Salids (Salt Plugs), Entisols (coarse-textured alluvial and colluvial soils, Regosols), and Lithic/Calcareous Haplocalcids (from gypsiferous and saliferous marls), reflecting high salinity, low organic matter, and calcareous content. The island has a hot desert climate (BWh, Köppen–Geiger) with very hot summers (mean > 33 °C), mild winters (~ 18–20 °C), and low annual precipitation (~ 150–160 mm, mostly in winter). These conditions provide the environmental context for the land suitability evaluation.

### Data collection and preparation

In this study, data were collected from various sources across Iran to analyze key environmental and geospatial factors. Geology data were obtained through geological maps and reports provided by national geological surveys, which explained in more details in Table 2. Soil science information was sourced from soil classification studies and fertility assessments conducted by local agricultural and environmental agencies. Water resources data, including hydrological patterns and Dams were gathered from regional water authorities and national water resource management organizations. Topographical information was derived from digital elevation models (DEMs) and topographic maps produced by the Iranian National Cartographic Center. LULC data were acquired through satellite imagery and LULC classification studies, ensuring comprehensive coverage of urban, agricultural, and natural areas. For the LULC mapping, we used Landsat imagery (was downloaded from the USGS EarthExplorer platform: https://earthexplorer.usgs.gov/) obtained on 06 June 2023, providing appropriate spatial coverage for accurate land-cover characterization. Information on natural hazards, such as floods and erosion was retrieved from disaster management agencies and seismic monitoring systems. Finally, environmentally sensitive areas were identified using conservation reports, biodiversity assessments, and ecological zoning studies conducted by Iranian environmental protection organization. These diverse datasets were integrated to provide a holistic understanding of the study region. All spatial data preparation, processing, and map generation were conducted using ArcGIS Desktop (ArcMap) version 10.8.2 (Esri, Redlands, CA, USA; https://www.esri.com/en-us/arcgis/products/arcgis-desktop/overview).

### Methodology

**Fig. 2:**
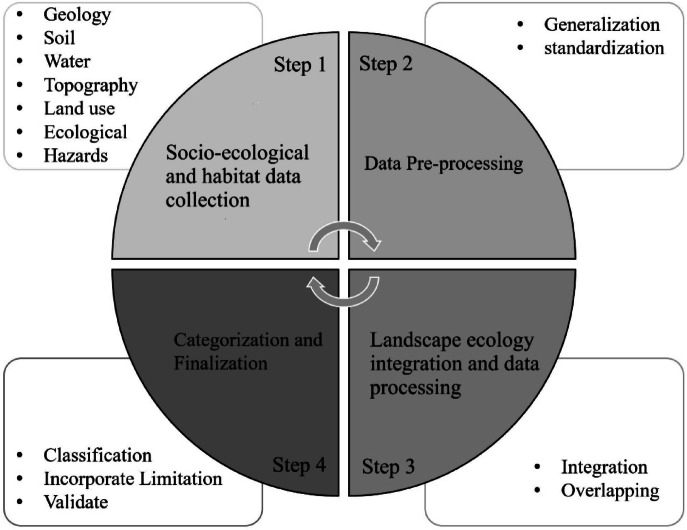
Overview of the land suitability assessment process: a four-step framework.

This study employed a systematic four-step process rooted in the landscape ecology approach, emphasizing the socio-ecological system, which integrates both ecological processes and human interactions within the landscape (Fig. 2). In the data collection phase, a diverse dataset was gathered, including geological, soil, water, topographical, LULC, ecological, and socio-environmental factors. Special attention was given to ecological components (e.g., habitat sensitivity, erosion risk, biodiversity hotspots) together with human-related spatial factors influencing agricultural development (e.g., existing LULC patterns, agricultural activities, and proximity to water resources). These datasets were sourced from reliable repositories to ensure accuracy and completeness.

During the data pre-processing phase, all collected data were standardized to facilitate their integration into a socio-ecological land suitability model^56–60^. Standardization ensured that both ecological gradients (e.g., slope stability) and human influences (e.g., land use intensity, proximity to settlements) were represented in a uniform spatial format.

The data processing phase involved integrating standardized criteria layers to capture the spatial dynamics of socio-ecological interactions^61^. Using advanced GIS-based modeling and spatial analysis techniques, the study analyzed how ecological constraints (e.g., biodiversity conservation zones, erosion-prone areas) interact with social factors (e.g., agricultural expansion, water access, infrastructure proximity)^62–65^. This approach provided a holistic perspective on land suitability by recognizing that landscapes are shaped by both natural processes and human interventions^36,66^.

Finally, in the categorization and finalization phase, the processed data were classified into suitability categories while considering both ecological integrity and socio-economic feasibility^57,65,67^. A limitation map was applied to exclude unsuitable areas due to ecological vulnerabilities or socio-economic constraints. The final suitability map was validated using ground-truth data and expert assessments, ensuring that the landscape ecology framework effectively guided agricultural planning^43,68^.

By integrating socio-ecological system principles, this methodology provides a comprehensive landscape-based framework that balances environmental conservation with agricultural sustainability, ensuring long-term ecological resilience and human well-being. Agricultural sustainability is described as the practice of managing agricultural systems in a way that maintains productivity while preserving ecological integrity.

In this study, the term “socio-ecological” refers to the integration of ecological conditions with human-related LULC and resource-use factors within a spatial planning framework. The study does not attempt to comprehensively model all socio-economic dimensions of agricultural systems, such as market accessibility, land tenure, or household economics, due to limitations in spatially explicit data availability.

### Model calibration

A new LULC planning model has been developed with an emphasis on agricultural capability, considering topographical factors, soil conditions, land characteristics, water accessibility, and ecological sensitivities. The goal of this model is to identify suitable and secure locations for agricultural intensification while accounting for the ecological sensitivity and capacity of the region. In today’s complex environment, decision-making and planning have become intricate processes due to the diversity and multitude of factors influencing them. To determine the suitability of landscape for various uses, ecological models have been created that allow for the assessment of land potential or capability for development activities^47^.

The ecological capability assessment model used in this study incorporates a range of factors specific to the region to achieve optimal and secure conditions for the allocation of LULCs. This model, originally developed by Makhdoom^47^, has been calibrated and applied in various regions in Iran based on their unique ecological conditions. For this study, the model was calibrated and applied based on the objectives, scale of the study, and the available data, ensuring that it is tailored to the specific characteristics of the study area.

The potential of the landscape for agriculture development is determined by two main factors (ecological and social) (Table 1). Additionally, there are inherent limitations to the development of this land use. Consequently, it is necessary to apply specific factors to impose appropriate restrictions on agricultural land use.

**Table 1:** Main criteria, factors, sub-criteria, and their weight and their fuzzy method in agricultural land use.

Main criteria	Weight	Factors	Sub-criteria	Standardization^47^	Sub-criteria Weight
Geology	0.09	Ecological	Geology	Geology fuzzy table (Table 2)	0.70
		Ecological	Geosite		0.30
Soil science	0.24	Ecological	Soil texture	Constant	1
Water resources	0.21	Ecological	Distance from waterways	Decreasing linear	0.20
		Social	Distance from dams	0-1000 meters = zero <br> 1000 meters and more = decreasing	0.30
		Ecological	Distance from shores	Decreasing Linear	0.5
Topography	0.14	Ecological	Slope	Decreasing Linear up to 15 percent	1
Land use	0.13	Social	Land use	Urban areas, Industrial areas, Mining, water bodies, Rock and Sandbars, and Tourism sites (0); Arid lands (0.2); forests (0.4); Grasslands (0.7); Pastures (0.8); Palm groves (1); Farmlands (1)	1
Natural hazards	0.07	Ecological	Distance from mangrove forests	Increasing linear	0.5
		Ecological	Flood risk	Constant	0.5
Ecologically sensitive areas	0.12	Ecological	Distance from mangrove forests	Increasing linear	0.45
		Ecological	Ecologically sensitive areas (Dolphins distribution, Turtle distribution, Turtle nesting)	Increasing linear	0.55

### Data pre-processing

#### Socio-ecological criteria generalization

The first step in evaluating the socio-ecological capacity of the landscape is to identify the factors effective in the capacity of landscape to support different land management plans, which was explained in the data collection section. After identifying the socio-ecological factors, which were based on availability and Reliability; Legal, Policy, and Environmental Requirements; Purpose of the Assessment (agricultural development); and finally Scale of Assessment (regional scale), it is necessary to standardize all the criteria^69^. For this purpose, all point and linear factors are first converted into distance maps and polygon factors converted into raster and assigned constant values to each class based on its suitability in the ArcGIS Desktop (ArcMap) version 10.8.2^70^. The layers are generated as a raster and the value of each pixel in the layer of each criterion is the value of the distance from the closest complication of the corresponding criterion^71^. We used standard raster format of the geology, geosite, soil texture, slope, LULC, erosion risk and distance layer from vector format data as waterways, dams, shores, mangrove forests, ecologically sensitive areas-dolphins distribution, turtle distribution, and nesting.

#### Socio-ecological criteria standardization

To achieve a standardized representation of the socio-ecological criteria, fuzzy logic was employed in the ArcMap, providing a robust framework for handling the inherent uncertainty and variability of environmental data^72^. Fuzzy logic enables the transformation of diverse criteria into comparable formats by assigning membership values to each pixel based on its distance from relevant features and based on its suitability^56,73,74^. This approach captures the gradual transitions and overlapping influences present in socio-ecological systems^59^. By applying fuzzy functions tailored to each criterion, such as linear, sigmoidal, or Gaussian functions (Tables 1 and 2), the data were normalized to a uniform scale, enhancing compatibility for multi-criteria decision-making^58,75–77^. This process ensured that the standardized map accurately reflected the spatial dynamics and interactions of the identified factors.

**Table 2:** Geological information of Qeshm Island.

Code	Geological class	Attribute	Fuzzy value
1	M2	Limestone and dolomtic limestone, in part organo-detritus; yellow and whitish (Guri Limestone Member/ lower Mishan Formation equivalent)	0.2
2	M3	Gray marl white thin argillaceous limestone intercalations	o.5
3	M4	Limestone marker bed, light yellow and whitish	0.3
4	M5	Gray marl with thin bedded sandy limestone intercalations	0.6
5	M6	Sandstone and marl, light brown and gray (transitional unit)	0.5
6	MP1	Sandstone, medium to thick bedded, moderately consolidated; brownwish cream and gray	0.7
7	P1	Marl with thin interactions of sandy marl; gray and brownwish gray	0.7
8	P1Q	Alternation of soft marly siltstone, sandstone and silty marl; light brown and gray (Bakhtyari Formation equivalent); usually coverd by marine terraces with transitional interval bed	0.8
9	PR-z	overburden of different ferruginous rock materials	0.3
10	Q	Superficial soil and silty mud	1
11	Q1	seasonal lake	0
12	Qa	Badland marly, sandy and silty alluvium	0.5
13	Qb	Beach sand and inland dunes	0
14	Qd	Prime beach sand dunes	0
15	Qg	Alluvial and gravel deposits; soft / unconsolidated sandstone and gritstone	1
16	QM	Mud flat	0
17	Qs	Sand sheet	0.2
18	Qt1	Terraces 01 to 07 (altitudes 220–151 m.)	0.9
19	Qt2	Terraces 08 to 11 (altitudes 150–101 m.)	0.9
20	Qt3	Terraces 12 to 17 (altitudes 100–51 m.)	0.9
21	Qt4	Terraces 18 to 28 (altitudes 50–00 m.)	1

### Data processing

#### Criteria weighting and aggregation

Following the standardization of socio-ecological criteria, the next step in the land suitability assessment involves criteria weighting and aggregation^60^. The Analytical Hierarchy Process (AHP) was employed to determine the relative importance of each factor, reflecting their significance in influencing land suitability^64,78^. AHP involves structuring the criteria into a hierarchical framework, comparing them pairwise, and calculating weights based on their relative importance. This method combines expert judgment and consistency checks to ensure the reliability of the assigned weights^79^. To obtain the expert judgments required for the pairwise comparisons, we distributed questionnaires to two expert groups (ten university professors with academic expertise in landscape ecology and sustainable land management and ten professionals working in the agricultural sector with practical field-based knowledge). Their evaluations were used to generate the pairwise comparison matrices using online Analytic Hierarchy Process (AHP) Tool (https://comcastsamples.github.io/ahp-tool/).

After weighting, the standardized criteria layers were aggregated to generate a composite suitability map^63^. This integration was performed using spatial analysis techniques in the ArcGIS Desktop (ArcMap) version 10.8.2, where the weighted layers were combined through a weighted overlay process^65,80^. The resulting map represents the synthesized impact of all criteria, highlighting areas with varying levels of suitability for the intended land use or management plans. The AHP-based approach ensures a systematic and transparent assessment, supporting informed decision-making for sustainable land management^81^.

#### Validation and sensitivity analysis

To ensure the accuracy and reliability of the generated suitability map, validation and sensitivity analysis were conducted^65^. Validation involves comparing the suitability map with existing LULC data, field observations, or expert knowledge to assess its alignment with real-world conditions using ArcMap (Spatial Comparison & Accuracy Assessment)^60^. Statistical metrics such as Kappa coefficient, Receiver Operating Characteristic (ROC) curve, or overall accuracy can be employed to quantify the model’s performance^62,82^. In this study, we used overall accuracy, because it effectively evaluates the model’s performance in our specific context.

In addition, sensitivity analysis was performed to evaluate the robustness of the results against variations in the assigned criteria weights. This process involved systematically altering the weights of individual criteria or groups of criteria and observing the changes in the suitability map. The sensitivity analysis helps identify the most influential criteria and ensures that the final results are not overly dependent on subjective weight assignments. Together, validation and sensitivity analysis enhance the credibility of the land suitability assessment and provide confidence in its application for decision-making.

## Results

### Socio-ecological criteria generalization and standardization

**Fig. 3:**
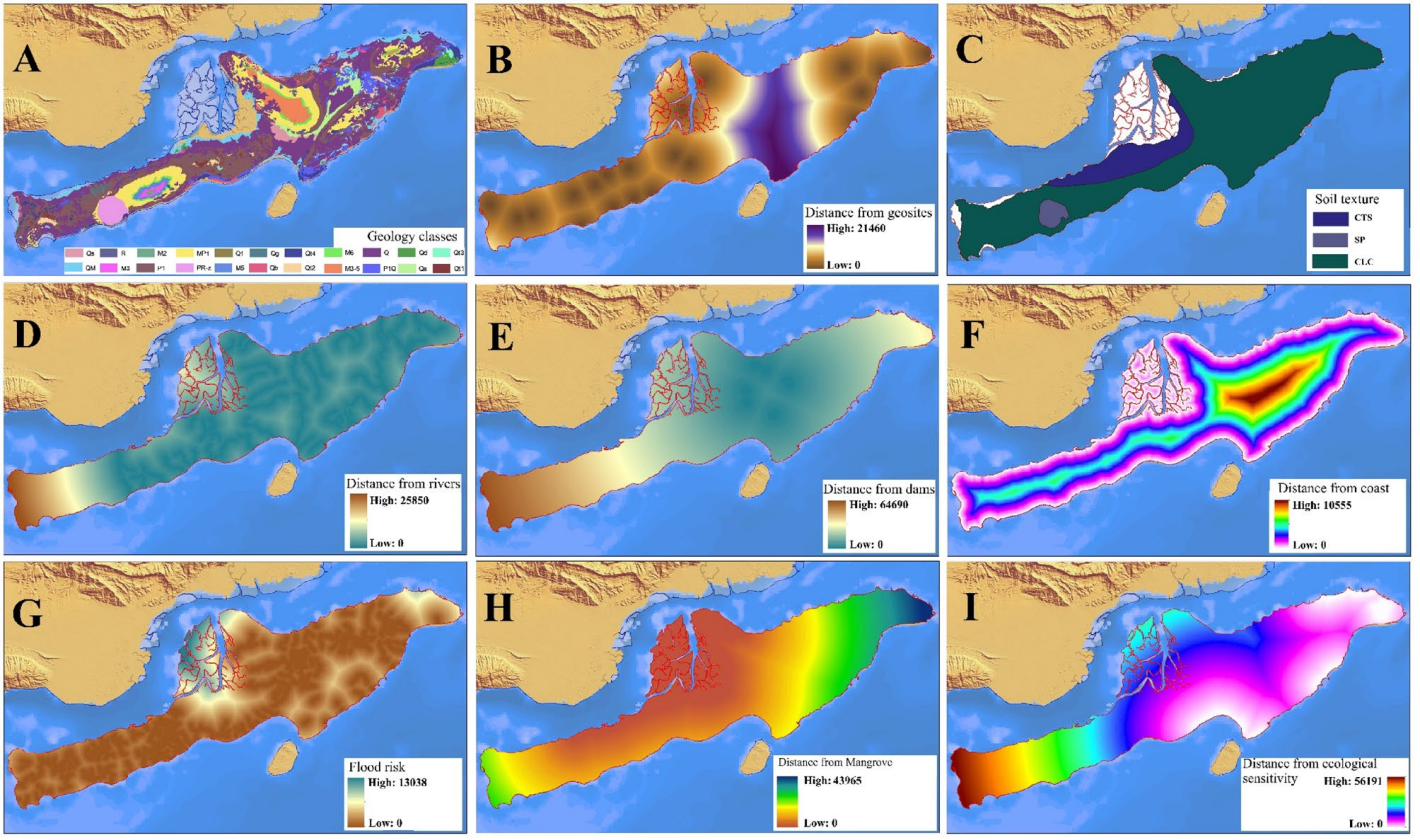
Generalized socio-ecological criteria. (**A**) Geology; (**B**) Geosites; (**C**) Soil texture; (**D**) Rivers; (**E**) Dams; (**F**) Coast; (**G**) Flood risk; (**H**) Mangrove forests; (**I**) Ecological sensitivity. Tree soil texture: CTS (Coarse Textured Soil with Alluvial, Colluvial, and Regosol soil type), SP (Salt Plugs), CLC (Calcareous Lithosols from saliferous and gypsiferous marls).

The socio-ecological criteria identified for the land suitability assessment were generalized and standardized to enable consistent comparison across heterogeneous datasets. The generalization process involved selecting key factors which have been presented in Fig. 3. These factors were derived from both raster and vector datasets, which were converted into uniform raster distance maps using the ArcGIS Desktop (ArcMap) version 10.8.2.

**Fig. 4:**
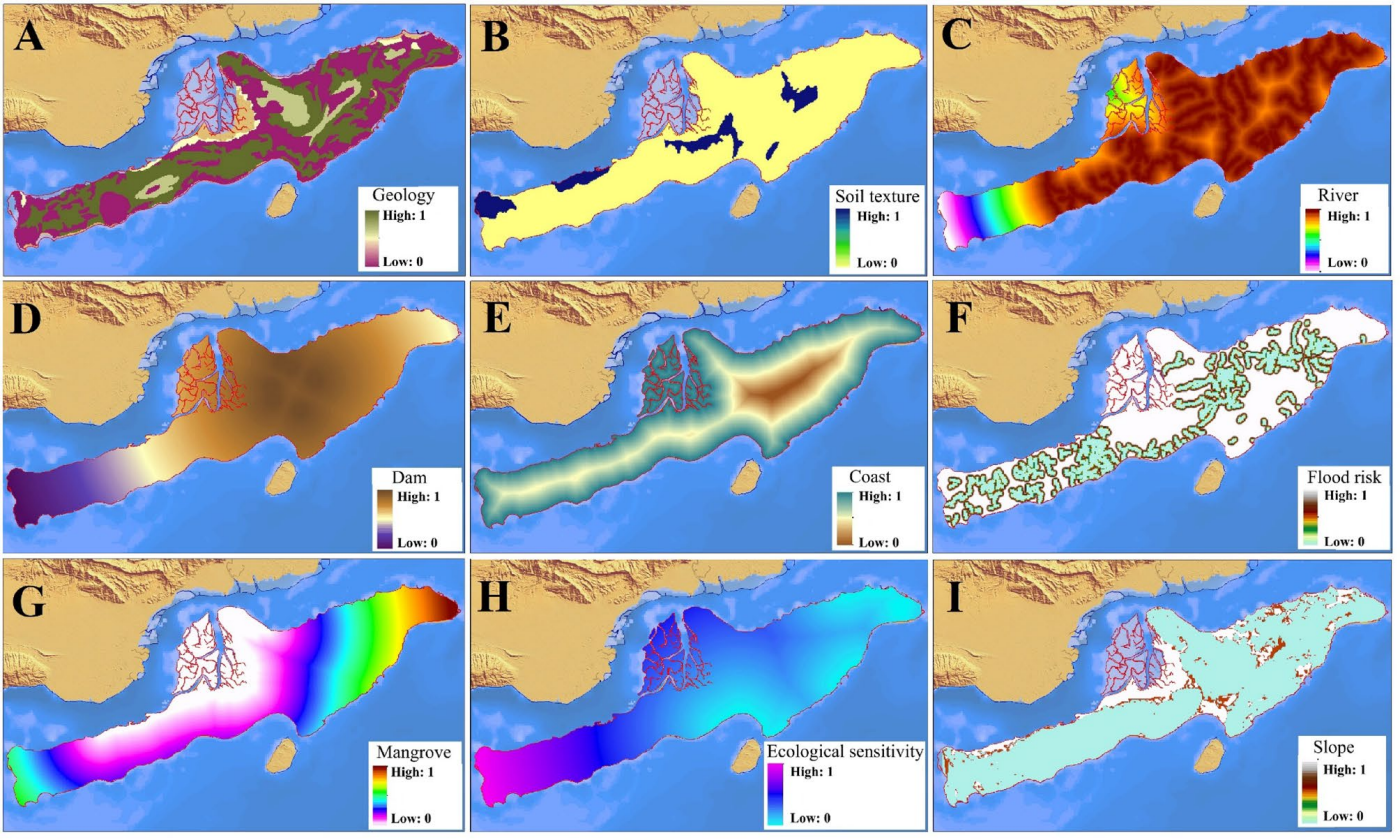
Standardized socio-ecological criteria. Fuzzy membership result as (**A**) Geology; (**B**) Soil texture; (**C**) Rivers; (**D**) Dams; (**E**) Coast; (**F**) Flood risk; (**G**) Mangrove forests; (**H**) Ecological sensitivity; (**I**) Slope.

All criteria layers were standardized by resampling them to a uniform spatial scale (pixel resolution) in ArcGIS and then fuzzy membership functions applied to normalize the data and account for scale differences and uncertainty across criteria. Fuzzy membership functions were applied to transform raw generalized data into a comparable spatial scale, where each pixel represented the degree of membership indicating suitability category between 0 and 1 (Fig. 4). This approach captured the continuous and overlapping nature of the socio-ecological factors, ensuring enhanced spatial fidelity of ecological gradients of their spatial dynamics. We minimized potential errors such as misalignment of raster grids, loss of detail from resampling, and uncertainty in boundary transitions by using the highest available resolution as the reference grid and applying nearest-neighbor interpolation, which ensures a more realistic representation of spatial dynamics and enhances the ecological consistency of the standardized layers.

### Composite suitability map and spatial patterns

**Fig. 5:**
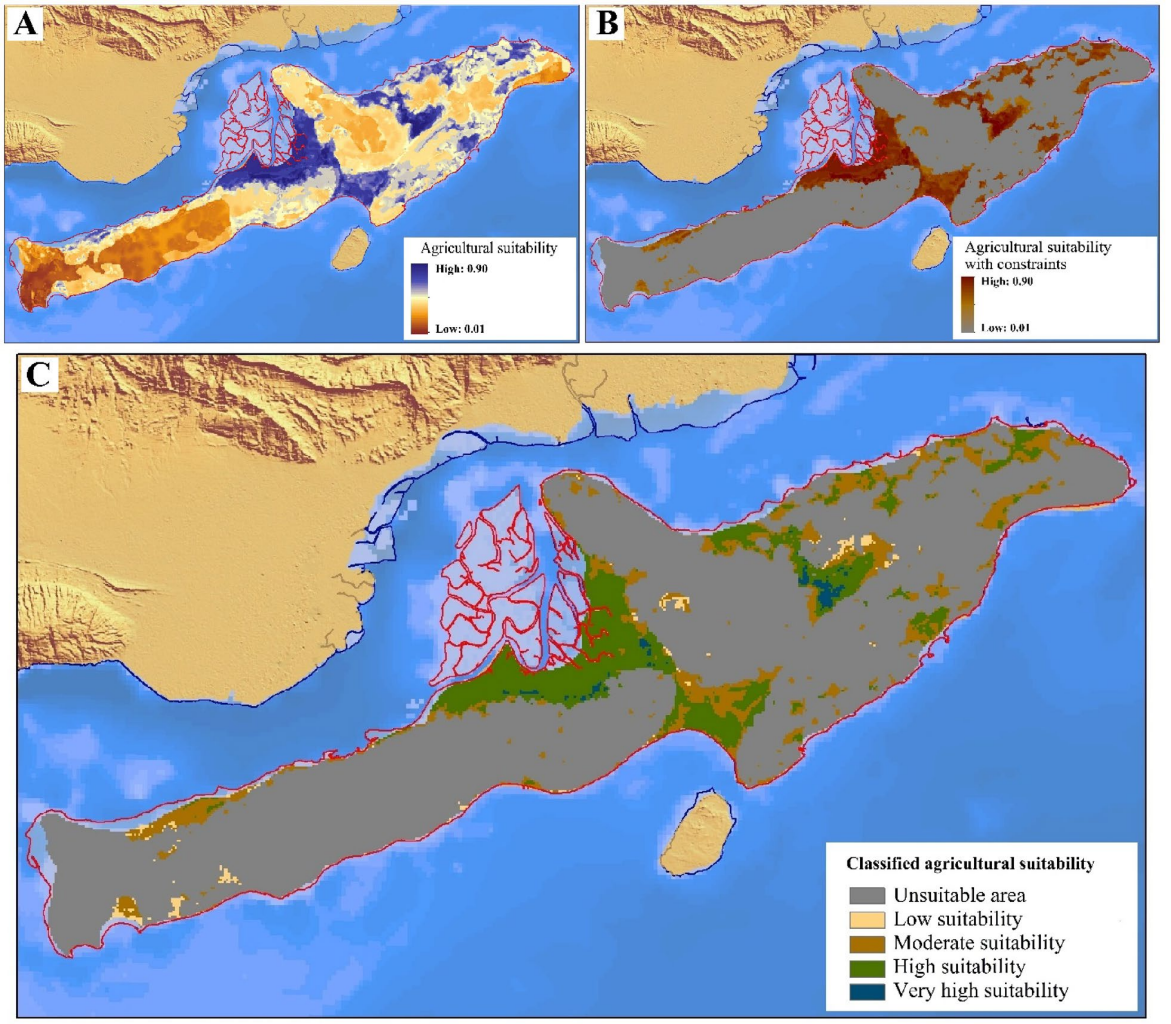
Spatial distribution of the landscape suitability assessment for agricultural land use. (**A**) Agricultural suitability without constraints (0.01–0.90 fuzzy value); (**B**) Agricultural suitability with constraints (0.01–0.90 fuzzy value); (**C**) Classified agricultural suitability.

The weighted aggregation of standardized socio-ecological criteria, using the Analytic Hierarchy Process (AHP) to assign relative importance, produced a composite suitability map, delineating areas of varying suitability across the study area. Figure 5A illustrates the agricultural development suitability map generated prior to exclusion of biophysical and ecological constraints. This preliminary map provides a general representation of the potential suitability of the landscape for agricultural development based solely on the weighted aggregation of socio-ecological criteria. The suitability values range continuously across the landscape, with scores from 0.01 to 0.90, highlighting areas with differing potential suitability.

To refine the analysis, exclusion criteria included steep slopes (> 15%), erosion-prone zones, and ecologically protected habitats were used to exclude unsuitable areas for agricultural development. Figure 5B presents the updated suitability map after incorporating these limiting criteria.

The final suitability map, shown in Fig. 5C, categorizes the landscape into five distinct classes based on fuzzy scores: unsuitable (0–0.2), low (0.2–0.4), moderate (0.4–0.6), high (0.6–0.8), and very high (0.8–1) suitability. This classification provides a clearer spatial interpretation of the results, enabling decision-makers to prioritize regions for agricultural development. Areas with very high suitability are concentrated in zones with optimal soil texture (Alluvial and Colluvial soils), gentle slopes, proximity to waterways, and favorable LULC patterns and cover a total of 812 hectares (Table 3). High-suitability zones encompass 17,816 hectares, were predominantly concentrated in regions with optimal geological features, low slope gradients, favorable soil textures, and proximity to critical socio-ecological resources such as waterways, mangrove forests, and key wildlife habitats.

**Table 3:** Area of the landscape suitability classes.

Landscape suitability classes	Area (ha)	%
Very high suitability	812	0.56
High suitability	17,816	12.19
Moderate suitability	13,935	9.54
Low suitability	1408	0.97
Unsuitable areas	112,188	76.74
SUM	146,159	100

Moderate-suitability areas (accounting for 13,935 hectares) met some but not all of the suitability thresholds for key criteria, often characterized by intermediate slope gradients or moderate distances from ecological resources. Low-suitability areas span 1,408 hectares were largely associated with steep slopes, high erosion risks, and significant distances from essential socio-ecological features. Unsuitable areas constitute the majority of the landscape, covering 112,188 hectares (Table 3). These zones are primarily influenced by severe limitations, including steep terrain, ecological sensitivities such as endangered species habitats, and distance from essential resources, making them inappropriate for agricultural development.

## Discussion

### Generalization and Standardization/ Integration of Socio-Ecological Factors

The process of generalizing and standardizing socio-ecological criteria laid a strong analytical foundation for the land suitability assessment that enabled consistent integration of both ecological and social dimensions of landscape. By converting diverse datasets (geology, soil, water, topography, natural hazards, Ecologically Sensitivity, and LULC datasets) into uniform raster layers, the study ensured spatial consistency and facilitated integration into the multi-criteria evaluation framework. As supported by Obiedat and Samarasinghe^72^, standardized raster layers are essential for reliable spatial modeling, enabling efficient weighting and aggregation processes, as further demonstrated in recent work by Fischer^59^.

The use of fuzzy logic membership functions to standardize the data proved particularly effective in managing the inherent uncertainty and variability across different ecological and physical parameters by allowing gradual, rather than binary, representation of parameter suitability^58^. This approach reflects the complex and continuous nature of real-world landscapes, where transitions between suitable and unsuitable areas are rarely abrupt^74^. The methodology preserved subtle spatial nuances such as gradual slope transitions, which would otherwise be lost in traditional binary classification systems, thereby producing more realistic and informative outcomes.

Although fragmentation and connectivity are widely recognized indicators in landscape-scale ecological assessments, the present study does not aim to perform a landscape metric analysis in the conventional sense. Instead, the study adopts a broader landscape ecology perspective focused on integrating ecological sensitivity, spatial heterogeneity, and human–environment interactions into agricultural land use planning. The framework therefore emphasizes landscape-level planning and socio-ecological integration rather than the quantitative measurement of landscape pattern indices alone.

### Spatial patterns and suitability interpretation

The spatial patterns derived from the composite suitability map reveal distinct spatial clusters of agricultural potential, as well as areas where development would be environmentally unsound. The introduction of exclusionary limitations, such as steep slopes, high erosion risk, and proximity to ecologically significant zones, substantially refined the assessment by marking areas as unsuitable for agriculture as mention in several studies^31,66,83^. This not only enhanced the ecological realism of the model but also provided guidance for land use zoning and resource allocation^61^. As Hobbs^84^ notes, identifying and preserving these zones is critical for preventing ecosystem degradation and supporting biodiversity conservation.

High-suitability zones correlate spatially with current agricultural land uses, lending empirical validity to the model. These areas, characterized by favorable slope gradients, soil texture, and proximity to water sources and wildlife habitats, represent optimal zones for sustainable intensification. In order to maximizing high-suitability zone’s agricultural potential, we need remedial actions. Sustainable intensification in the study of Pretty^85^ refers to remedial actions, which refers to agricultural systems and practices that maintain or increase production while delivering significant improvements in environmental outcomes. It emphasizes boosting system performance without expanding cultivated land or causing the loss of natural habitats, and without imposing any net environmental cost^85–89^. This action emphasizes vertical development through intensification, technological upgrading, and improved management practices within existing agricultural lands^87^. Moderate-suitability areas, covering 13,935 hectares, exhibit partial compliance with critical criteria. These landscapes may be viable for agriculture with targeted interventions (e.g., terracing, drip irrigation, and soil fertilization) to mitigate existing limitations^90^. Their intermediate suitability scores suggests that policy or technical investments could convert them into high-potential zones with minimal environmental cost which mentioned in several studies^14,90,91^.

Low-suitability areas (1,408 hectares) are often associated with steep topography, erosion-prone soils, and remoteness from essential ecological features. These zones are recommended for low-impact uses, including conservation buffer zones, biodiversity corridors, agroforestry, or ecotourism^92,93^. Their preservation is vital to maintaining ecological function and preventing land degradation.

Unsuitable areas, encompassing 112,188 hectares, were defined by severe physical and ecological constraints. These include steep slopes, highly erodible soils, and proximity to ecologically sensitive habitats such as mangrove forests and turtle nesting sites. These zones are clearly incompatible with agricultural expansion due to irreversible limitations and should be prioritized for conservation or regulated land use to uphold long-term ecosystem services^94^.

maximizing the production potential in the Low and unsuitable areas requires enhancement actions, which refers to targeted interventions aimed at improving the biophysical conditions of the landscape, such as soil rehabilitation, salinity mitigation^95,96^, erosion control, and water infrastructure development^97^, which enables limited horizontal agricultural development where environmental constraints can be realistically reduced.

### Methodological and policy implications

The integration of biophysical with socio-economic criteria advances an ecosystem-based land use planning framework to landscape planning^25,61^. The results support evidence-based decision-making at regional and local scales, enabling stakeholders to balance development goals with ecological preservation. This approach is consistent with international frameworks such as the Convention on Biological Diversity^98^ and the UN Sustainable Development Goals^99^, which emphasize ecosystem-based land management.

Furthermore, the delineation of suitability classes facilitates prioritization of land for development, restoration, or protection. Planners can use this suitability maps and spatial overlays to direct agricultural investments to high-potential zones, while simultaneously safeguarding fragile ecosystems from unsustainable conversion. By embedding enhancement and remedial strategies within a suitability-based framework, policymakers can ensure that both expansion and intensification are applied selectively, efficiently, and sustainably, thereby minimizing ecological risks while optimizing the island’s agricultural productivity^89^.

For lands classified as low suitability or currently unsuitable, policy should prioritize carefully targeted enhancement measures aimed at improving biophysical constraints where feasible. These interventions may include soil rehabilitation, salinity mitigation, erosion control, and the development of water infrastructure. Such actions can enable limited and controlled horizontal agricultural development in areas where environmental limitations can be realistically reduced. However, this pathway must be applied selectively, based on environmental feasibility assessments and cost–benefit evaluations, to avoid ecologically or economically unsustainable expansion^96,97^. In contrast, medium and high suitability landscapes should be governed by policies that promote vertical development rather than spatial expansion. Remedial actions in these zones should focus on sustainable intensification through technological upgrading, improved farm management practices, input optimization, and productivity-enhancing innovations within existing agricultural boundaries^100^. This approach strengthens output capacity while minimizing pressure on natural ecosystems and reducing the need for land conversion^85–89^.

### Limitations and recommendations for future work

While the methodological framework is comprehensive, a few limitations merit acknowledgment. First, the analysis is based on static spatial and single-year LULC data, which does not account for temporal changes in LULC, climate, or ecological dynamics. Incorporating time-series data and climate projections could improve the predictive accuracy and adaptability of the model. Second, the weighting of criteria was derived from expert judgment and literature, which may reflect disciplinary bias or contextual limitations. Future research could employ participatory approaches, such as stakeholder engagement (e.g., local farmers and conservation planners) to increase transparency and contextual relevance. Lastly, while socio-ecological variables were well-represented, socio-economic drivers such as population density, land tenure, market access, infrastructure availability, and local economic conditions were not included due to limited availability of reliable spatially explicit data. Their integration could enhance the practical applicability of the model, especially in policy or development planning contexts.

## Conclusions

This study developed a standardized framework for land suitability assessment by integrating socio-ecological criteria, such as slope and proximity to sensitive habitats and essential resources, using fuzzy logic, AHP-based weighting, and GIS-based spatial analysis. The resulting composite suitability map spatially differentiates areas with varying agricultural potential from unsuitable to very high suitability, highlighting the influence of key environmental factors such as soil texture, slope, proximity to water resources, and ecological sensitivities (presence of mangrove forests and turtle nesting sites). The classification into five suitability levels provides a structured spatial interpretation of land potential, enabling informed decision-making for sustainable land use planning for planners and policymakers.

Quantitatively, the analysis shows that 12.75% of the island falls within the high and very high suitability classes, primarily in areas characterized by favorable slope gradients, soil texture, proximity to water sources, and favorable distance to sensitive wildlife habitats. In contrast, 77.71% of the island is classified as low or unsuitable for cultivation, strongly influenced by steep topography, erosion-prone soils, remoteness from essential ecological features, and proximity to ecologically sensitive habitats such as mangrove forests or turtle nesting sites.

The weighting results highlight the relative importance of key socio-ecological criteria: soil texture and slope received the highest influence values, followed by distance to water resources, while ecological sensitivity layers (mangroves, turtle habitats) acted mostly as exclusionary constraints, reducing suitability scores by 15–25% in areas with high conservation value. These findings demonstrate that integrating biophysical and ecological layers substantially alters the spatial configuration of suitable land, compared with traditional models that rely solely on physical parameters. The findings emphasize the importance of considering both favorable conditions and limiting factors in land suitability assessments to ensure environmentally responsible agricultural expansion.

In this study, maximizing the island’s agricultural potential is understood through two distinct but complementary management pathways: enhancement and remedial actions, each aligned with different suitability classes. Enhancement refers to targeted interventions aimed at improving the biophysical conditions of currently low-suitability or unsuitable lands, such as soil rehabilitation, salinity mitigation, erosion control, and water infrastructure development, which enables limited horizontal agricultural development where environmental constraints can be realistically reduced. In contrast, remedial actions focus on medium to high suitability areas, emphasizing vertical development through intensification, technological upgrading, and improved management practices within existing agricultural lands to increase productivity without further land expansion. This differentiated strategy allows agricultural development to be spatially guided by suitability levels, ensuring that expansion and intensification are applied selectively and sustainably while minimizing ecological risks.

From a planning perspective, high and very high suitability zones present opportunities for integrated cultivation, while low and unsuitable areas should be designated for conservation or low-impact uses to prevent ecological degradation. This quantitative differentiation supports more precise land use zoning and provides spatial guidance for policymakers in balancing agricultural development with biodiversity conservation, promoting sustainable resource management across Qeshm Island. Overall, the study demonstrates that integrating ecological sensitivity, land-use dynamics, and human-related spatial factors within a landscape ecology-based planning framework can produce more ecologically informed land suitability outcomes and provide a replicable decision-support tool for sustainable agricultural planning in scare-resource landscapes.

## Data Availability

The datasets generated and/or analysed during the current study are available from the corresponding author on request.
